# A partial deletion within the meiosis-specific sporulation domain *SPO22* of *Tex11* is not associated with infertility in mice

**DOI:** 10.1371/journal.pone.0309974

**Published:** 2024-09-04

**Authors:** Farah Ghieh, Bruno Passet, Elodie Poumerol, Johan Castille, Pierre Calvel, Jean-Luc Vilotte, Eli Sellem, Luc Jouneau, Hendrick Mambu-Mambueni, Henri-Jean Garchon, Eric Pailhoux, François Vialard, Béatrice Mandon-Pépin

**Affiliations:** 1 UVSQ, INRAE, BREED, Université Paris-Saclay, Jouy-en-Josas, France; 2 öcole Nationale Vétérinaire d’Alfort, BREED, Maisons-Alfort, France; 3 INRAE, AgroParisTech, GABI, Université Paris Saclay, Jouy-en-Josas, France; 4 R&D Department, ALLICE/Eliance, Paris, France; 5 UMR1179, UVSQ, Montigny le Bretonneux, France; 6 Département de Génétique, Laboratoire de Biologie Médicale, CHI de Poissy/Saint- Germain-en-Laye, Poissy, France; Universite Clermont Auvergne, FRANCE

## Abstract

Azoospermia (the complete absence of spermatozoa in the semen) is a common cause of male infertility. The etiology of azoospermia is poorly understood. Whole-genome analysis of azoospermic men has identified a number of candidate genes, such as the X-linked testis-expressed 11 (*TEX11*) gene. Using a comparative genomic hybridization array, an exonic deletion (exons 10–12) of *TEX11* had previously been identified in two non-apparent azoospermic patients. However, the putative impact of this genetic alteration on spermatogenesis and the azoospermia phenotype had not been validated functionally. We therefore used a CRISPR/Cas9 system to generate a mouse model (*Tex11*^*Ex9-11*del/Y^) with a partial *TEX11* deletion that mimicked the human mutation. Surprisingly, the mutant male *Tex11*^*Ex9-11*del/Y^ mice were fertile. The sperm concentration, motility, and morphology were normal. Similarly, the mutant mouse line’s testis transcriptome was normal, and the expression of spermatogenesis genes was not altered. These results suggest that the mouse equivalent of the partial deletion observed in two infertile male with azoospermia has no impact on spermatogenesis or fertility in mice, at least of a FVB/N genetic background and until 10 months of age. Mimicking a human mutation does not necessarily lead to the same human phenotype in mice, highlighting significant differences species.

## Introduction

Infertility is a significant health problem and affects around 7% of men of reproductive age [1]. About 10% of infertile men cannot conceive due to a complete absence of sperm in their ejaculate. This severe male infertility phenotype is referred to as azoospermia and can result from primary testicular failure (non-obstructive azoospermia (NOA)) or obstruction of the ejaculatory ducts (obstructive azoospermia (OA)). By using assisted reproductive technologies (ARTs, such as testicular sperm extraction (TESE)), men with OA may be able to father children if spermatozoa are found in the testes. However, no spermatozoa can be retrieved in cases of complete germ cell meiotic arrest or Sertoli cell-only syndrome (SCOS), and so other options (such as sperm donation and adoption) must be considered.

Chromosome aberrations can explain the NOA observed in some individuals (for review, see [2]). However, the etiology and pathophysiology of NOA are not known for the significant proportion of infertile men with no chromosome aberrations. In some of these individuals, azoospermia might be due to single-gene variations. Indeed, a recent review of genome-wide studies listed over 50 genes linked to human NOA [1, 3]. Several (including *STAG3*, *SYCE1*, and *TEX11*) have been reported in more than one study [2, 3].

The X-linked testis-expressed gene 11 (*Tex11*) is required for meiotic recombination and chromosomal synapsis in mice [4, 5]. Tex11 was identified as one of the X-linked testis-specific genes in a genomic screen [6].Alterations in *TEX11* were first described using high-resolution comparative genomic hybridization, with the loss of three exons (exons 10–12; (c.652del237bp)) in two azoospermic patients (one with mixed testicular atrophy and one with a meiotic arrest phenotype) [7]. This exonic loss does not generate a frameshift but induces a 79-amino-acid deletion within the protein’s meiosis-specific sporulation domain SPO22. In the same report, screening of a series of 289 azoospermic men revealed five other variants in the *TEX11* open reading frame (three meiotic arrests, one mixed testicular atrophy, and one partial meiotic arrest). In another study, a splicing acceptor site mutation and a number of missense mutations were also identified in human *TEX11* gene of azoospermic men [8]. Since these two studies [7, 8], *TEX11* mutations have been reported in the literature on a regular basis [8–16]. The discovery of *TEX11* mutations has stimulated interest in the underlying causes of azoospermia in general and of meiotic arrest in particular. Usefully, *Tex11* knock-out (KO) mice have an azoospermic phenotype similar than to seen in patients [4].

However, most gene variants associated with sperm maturation arrest have not been validated in cell-based models or animal models; only *in silico* analyses or non-functional investigations were used to assign the variant’s pathogenicity. *In silico* prediction tools should be used with caution, and recent research has shown that *in vivo* modeling of the human variant alone does not always predict the phenotype seen in mice [17].

To date, only two transgenic mice mimicking human *TEX11* mutations have been described [8, 18]. The validation of these mutations in humans is essential for understanding the mechanisms of azoospermia and for developing innovative therapeutic solutions. Hence, with a view to evaluating the causative role of the recurrent deletion of the three *TEX11* exons (c.652del237bp) in the observed azoospermic phenotype and studying the mutation’s possible pathogenic role, we used a clustered regularly interspaced short palindromic repeats/CRISPR-associated protein 9 (CRISPR/Cas9) technique to generate a mutant mouse line (*Tex11*^*Ex9-11*del/Y^) carrying an equivalent exonic deletion.

## Materials and methods

### Ethics statement

All animal experiments were performed in strict accordance with the European Union’s Code for Methods and Welfare Considerations in Behavioral Research with Animals (Directive 2016/63/UE). All experiments were approved by the Animal Care and Use Committee for Experiments at the National Research Institute for Agriculture, Food and Environment (INRAE) (COMETHEA, Jouy-en-Josas, France; reference: 18–12) and had been authorized by the French Ministry for Higher Education, Research and Innovation (reference: 815–2015073014516635).

### Mice

**Fig 1:**
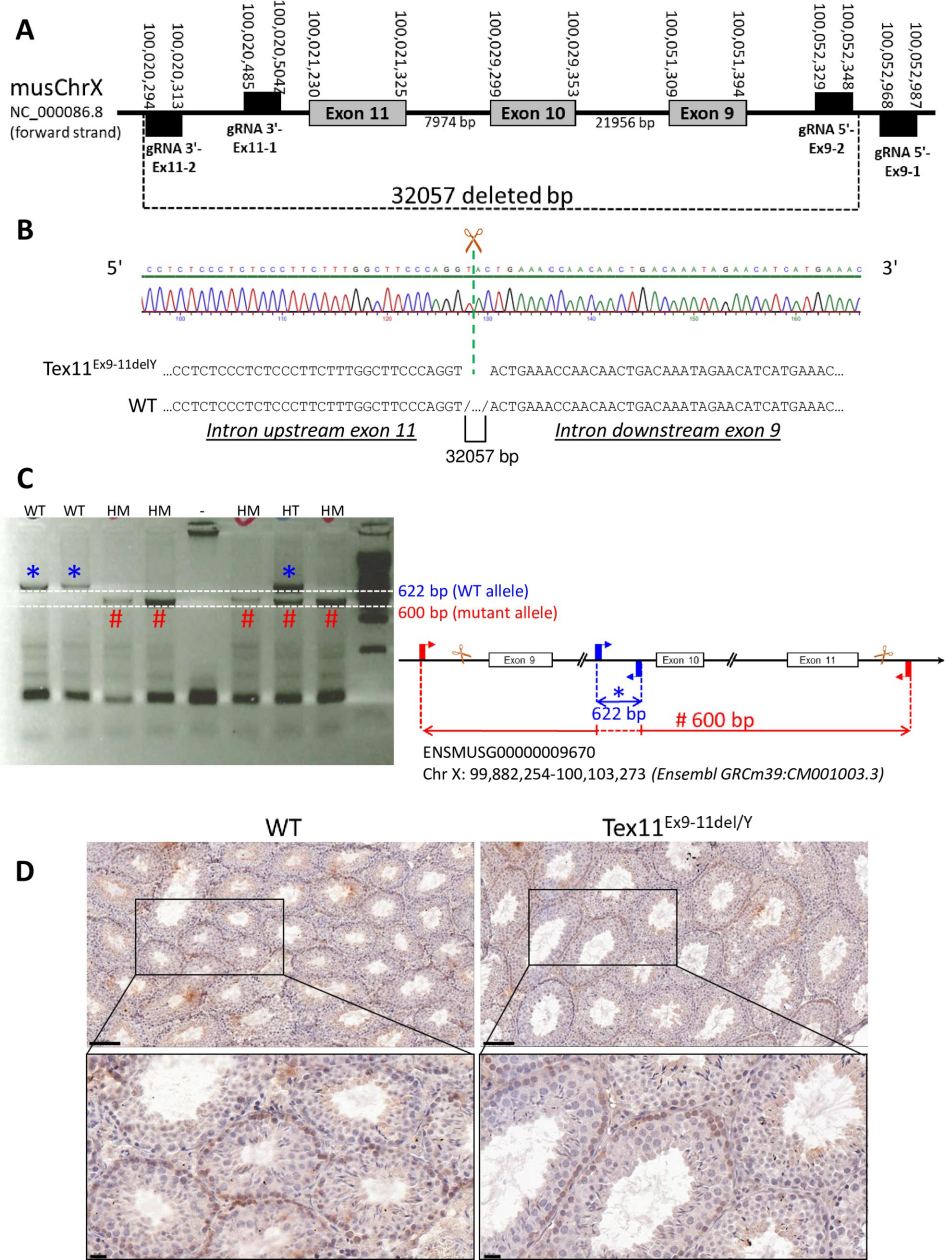
Generation of *Tex11*^Ex9-11del/Y^ mutant mice. (A) A schematic representation of CRISPR/Cas9 deletion of the *Tex11* gene, with the removal of three exons (e9, e10, e11), two introns (i9, i10), and parts of the introns (i8, i11) either side of exons 9 and 10. Grey boxes correspond to deleted exons and lines represent introns. (B) Electropherogram of genomic DNA from the *Tex11*^*Ex9-11*del/Y^ mouse, showing a 32057 bp deletion that encompassed exons 9 to 11. (C) PCR genotyping of DNA from *Tex11*^*Ex9-11*del/Y^ mutant mice (left panel). HM: hemizygous male mutant mouse; HT: heterozygous female mutant mouse. A schematic representation of the size of the PCR products obtained (right panel). The primers used for PCR genotyping of WT (blue) and TEX11 (red) mice are shown. (D) TEX11 location on mouse adult WT and *Tex11*^*Ex9-11*del/Y^ testes by immunostaining. Positive signal for TEX11 is visible as brown precipitate and counterstain was realized with hematoxylin. The positive signal is localized to the periphery of certain tubules and observed the cytoplasm and nuclei of some spermatogonia and early spermatocytes of WT and *Tex11*^*Ex9-11*del/Y^ testes. Scale bars: 100 μm (top) and 20 μm (bottom).

The mutant *Tex11* transgenic mouse line *Tex11*^*Ex9-11*del/Y^ was generated using CRISPR/Cas9 genome editing technology. The RNA mix comprised an mRNA coding for SpCas9-HF1 nuclease and the four single guide RNAs (sgRNAs) targeting exons 9 to 11 of the *Tex11* gene (NC_000086; NM_031384). Using CRISPOR software (http://crispor.tefor.net/), we designed the sgRNAs to remove the exons 9, 10, and 11, introns 9 and 10, and parts of introns 8 and 11. The sgRNA sequences and locations are shown in S1 Table and Fig 1, respectively.

The Cas9-encoding mRNA and the four sgRNAs were injected (100 ng/μL for each) into single-cell fertilized FVB/N mouse eggs [19]. The surviving injected eggs were transferred into two pseudopregnant recipient B6CBA/F1 mice (23 eggs per mouse). To establish transgenic lines, the transgenic founder mice were crossed with wild-type (WT) FVB/N mice. F1 heterozygous mice in each line were crossed together to obtain F2 hemizygous male mice or homozygous female mice and thus establish the *Tex11*^*Ex9-11*del/Y^ mouse lines. Once the line had been established, all pups were genotyped with a combination of primers (listed in S1 Table). The *Tex11*^*Ex9-11*del/Y^ mutant mice were fertile and grew normally.

All mice were housed at the UE0907 facility (INRAE, Jouy-en-Josas, France) at a temperature of 25°C and with a 12 h/12 h light/dark cycle and *ad libitum* access to food. With regard to the 3Rs and improving receptiveness, the animals were placed in an enriched environment.

### Mouse genotyping

Genomic DNA was obtained from tail biopsy specimens of the resulting live pups. The samples were genotyped by PCR amplification of *Tex11* exons 9, 10, and 11 (see S1 Table for the genotyping oligonucleotide sequences). Two sets of primers were used: one that amplified 622 bp to check the WT allele (primers Tex11_64241F and Tex11_64843R), and one that only amplified 600 bp when the exonic deletion was present (primers Tex11_50129F and Tex11_83418R). The exonic deletion was confirmed by sequencing the amplified genomic fragment. The genotyping PCR was performed according to the manufacturer’s instructions, using the TaKaRa Ex Taq® DNA Polymerase kit (Takara Bio, San Jose, CA, USA). The PCR conditions were 35 amplification cycles with 94°C for 30 s, 60°C for 30 s and 72°C for 30 s.

### Evaluation of the mice’s fertility

Upon sexual maturation, F2 hemizygous male mutant mice were caged individually with two 8-week-old WT FVB/N female mice for 6 months. After the mating period, male mice were removed from the cages. Breeding cages were monitored daily. Gestations, birth dates, and litter sizes were recorded. The number of pups born was recorded, and the average litter size for each mouse line was calculated. The litter size per female was recorded for 6 months.

### Testicular and sperm analyses for mutant male mice

After the fertility test, adult mutant male mice and same-aged WT FVB/N mice were euthanized by cervical dislocation. We recorded the testis weights, testicular histology, and sperm variables. Testis tissues were dissected, measured for weight and then fixed for histological analysis (as indicated below) or flash frozen immediately in liquid nitrogen before storage at -80°C. The frozen tissues were used for the transcriptomic and molecular analyses described below. Cauda epididymis were also removed from mutant and WT mice and weighed.

Sperm concentration and motility (including motile spermatozoa, progressive spermatozoa, average path velocity (V_AP_), progressive velocity (V_SL_), and the track speed (V_CL_)) were recorded in 8-week-old mutant *Tex11*^*Ex9-11*del/Y^ and WT mice using the Computer-Assisted Sperm Analysis (CASA) (Hamilton Thorne Inc., Beverly, MA, USA). Cauda epididymis from each mouse was removed, immediately plunged in 100 μl Tris, citrate and fructose buffer and cut up with scissors to release the spermatozoa. The samples were then incubated for 10 minutes at 37°C, in order to collect the spermatozoa. A 4 μl aliquot was placed on a standardized, four-chamber Leja counting slide (Leja products B.V., Nieuw-Vennep, the Netherlands). Ten microscope fields were analyzed on an automated stage, using the predetermined starting position within each chamber. The ten fields (containing at least 300 cells) were analyzed statistically. The protocol and parameters used have already been described [20].

### Histological analyses

For histological studies, fresh testicular and epididymal tissues from 8-week-old mice (n = 3 by genotype) were fixed in 4% paraformaldehyde (Electron Microscopy Sciences, reference 50-980-495) in phosphate-buffered saline (PBS) at 4°C. After rinsing in PBS, the tissues were stored in 70% ethanol at 4°C. The tissues were included in paraffin using a Citadel automat (Thermo Fisher Scientific Shandon Citadel 1000, Waltham, MA, USA) and a standard protocol.

Paraffinized tissue sections (thickness: 4 μm) were prepared and sorted on a Superfrost Plus Slides system (reference J1800AMNZ, Thermo Fisher Scientific). Once dry, the slides were stored at 4°C.

For the histologic analyses, testes sections were stained with hematoxylin-eosin reagent at the @Bridge facility (INRAE, Jouy-en-Josas, France), using an automatic Varistain Slide Stainer (Thermo Fisher Scientific). Periodic acid-Schiff staining was used to determine the seminiferous epithelium stages in the testes.

### Immunohistochemistry analyses

Tissue sections were deparaffinized for 10 minutes at 60°C and then rehydrated in successive baths of xylene and ethanol at room temperature. Firstly, slides were exposed to a citrate buffer (pH 6.0; H-3300, Vector Laboratories). After inactivation of endogenous peroxidase activity with a 0.3% H2O2 solution (H1009, Sigma-Aldrich), section were blocked with 2.5% normal horse serum (from ImmPRESS anti-goat kit MP-7405, Vector Laboratories) for 30 minutes at room temperature. Primary antibody (anti-TEX11, reference AF5627, R&D Systems, immunogen: Met768-Leu947—the last 180 amino acids of the mouse TEX11 protein) was then applied (dilution 1/20) and incubated for 1 hour and half at 37°C. Following washes with PBS 1x, slides were subject to secondary antibody (supplied in ImmPRESS kit) for 30 minutes at room temperature. Finally, the antibody was revealed with DAB enzymatic reaction (10 min, SK-4100, Vector Laboratories), followed by brief hematoxylin counterstaining (5 sec). The slides were then dehydrated and mounted with Eukit. Stained sections were scanned with a 3DHISTECH panoramic scanner at the @Bridge facility (INRAE, Jouy-en-Josas, France).

### Total RNA extraction

Total RNA was isolated from the testes of four- and five-month-old mice, using TRIzol reagent (Invitrogen, 15596–026). The RNA was then purified using the RNeasy Mini kit (Qiagen, CA, USA), according to the manufacturer’s instructions. The total RNAs were treated with DNase to remove DNA (RNase Free DNase Set, 79254, Qiagen). The quality of the extracted total RNA was checked on a 2100 BioAnalyzer (Matriks, Norway) and quantified in a Qubit® fluorometric assay (Thermo Fisher Scientific).

### RNA sequencing

Samples with an RNA integrity number >8 were deemed to be suitable for RNA sequencing (RNA-seq) (WT n = 3; *Tex11*^*Ex9-11*del/Y^ n = 9). Stranded library preparation (New England Biolabs Preparation Kit, NEB #E7760, #E7765) and sequencing (paired-end 2x75 cycles; NextSeq 500 Illumina) were performed at the University of Versailles Saint-Quentin-en-Yvelines (UVSQ) GenΩmics high-throughput sequencing facility (https://www.sante.uvsq.fr/plateforme-de-genomique-genomics). Twenty-seven to 40 million fragments were generated per sample. The results were demultiplexed (bcl2fastq2-2.18.12), and the adapters were removed (Cutadapt1.15). Only reads longer than 10 bp were analyzed. FastQC v0.11.5 was used to check the quality of the raw RNA-seq data.

### Transcriptomic analysis

Sequence libraries were aligned with the Ensembl 99 genome using TopHat [21], and gene table counts were obtained by applying featureCounts to these alignments [22]. The data were normalized and the single-gene differential expression levels were analyzed using DESeq2 [23]. The threshold for statistical significance in a Benjamini-Hochberg test was set to an adjusted p<0.05 and an absolute log2 fold-change >1 [24]. Raw RNA-seq data were deposited via the SRA Submission portal (https://submit.ncbi.nlm.nih.gov/subs/sra/) (BioProject ID PRJNA1015668).

### Statistics

For each experiment, median and interquartile values were plotted using GraphPad Prism, and statistical analyses were performed with KrusKall–Wallis tests under R software [Rcmdr package].

### Modeling TEX11

Structural TEX11 models were generated with the Phyre2 algorithm [25] (http://www.sbg.bio.ic.ac.uk/phyre2/html/page.cgi?id=index). To visualize the three-dimensional (3D) structure of TEX11, we used the PyMOL software (https://pymol.org/2/) [26].

## Results

### *Tex11* targeting and deletion in the mouse

To mimic the human deletion comprising exons 10 to 12 of *TEX11* isoform 1 (NM_001003811.1) identified in azoospermic patients with meiotic arrest [7], we targeted the mouse exons 9–11 (NM_031384) (Fig 1A).

We first compared the *Tex11* and *TEX11* transcripts (S2 Table and S1A Fig). In an analysis of the BLAST-aligned nucleotide sequences, we found that the human (NM_001003811.1) and mouse (NM_031384) mRNAs were about 75% homologous (cover 86%). The unaligned regions corresponded to (i) the first three human exons and first two murine exons, and (ii) the last exon (human exon 31 and mouse exon 30) (S1B Fig and S2 Table). The proteins corresponding to these transcripts were also aligned with using Blastp (NP_001003811.1 and NP_113561.2, respectively, for humans and mice) (S1C Fig). Due to the presence of a supernumerary exon 3 in humans, human exons 10–12 are homologous to murine exons 9–11. Thus, the human deletion comprising exons 10, 11 and 12 of NM_001003811.1 was mimicked by the genomic deletion comprising murine exons 9, 10 and 11 of NM_031384 (see S2 Fig for sequence homology between human and murine exons of interest).

To cover the area of interest, two sgRNAs were located 5’ to murine exon 9 and two sgRNAs were located 3’ to exon 11, in order to target the full length of exons 9–11 (Fig 1A) and to improve the cutting efficiency [27].

Mouse founders with a disrupted target site were identified by Sanger sequencing of the PCR-amplified region surrounding the deleted exons (Fig 1B). The *Tex11*^*Ex9-11*del/Y^ mutant mouse line had a 32,057-base deletion of exons 9 to 11, introns 9 and 10, and parts of the immediately upstream and downstream introns (8 and 11, respectively). The *Tex11* intragenic deletions of exons 9 to 11 in mice were confirmed using PCR analysis (Fig 1C).

### Expression of the TEX11 protein in testes

The deletion produced a truncated TEX11 protein in mutant mice; there was no frameshift, and the protein was 79 amino acids shorter than the WT. We used an immunohistochemistry assay to confirm the expression of truncated TEX11 in the testes of *Tex11*^*Ex9-11del/Y*^ mice (Fig 1D). This analysis of testis sections (using an antibody targeting the C-terminal part of the murine protein; exons 27 to 30) indicated that relative to littermate controls, TEX11 protein was expressed normally in the *Tex11*^del/Y^ mutants and localized to the periphery of certain tubules. The positive signal is localized in the cytoplasm and nuclei of some spermatogonia and early spermatocytes in WT and *Tex11*^*Ex9-11*del/Y^ mice (Fig 1D), in agreement with previous studies of the expression profile of TEX11 in wild-type mice [4, 5, 7]. Hence, the exonic deletion did not affect the expression of a shorter TEX11 protein in mutant mice.

### Targeted exonic deletion of Tex11 did not affect the mice’s fertility

**Fig 2:**
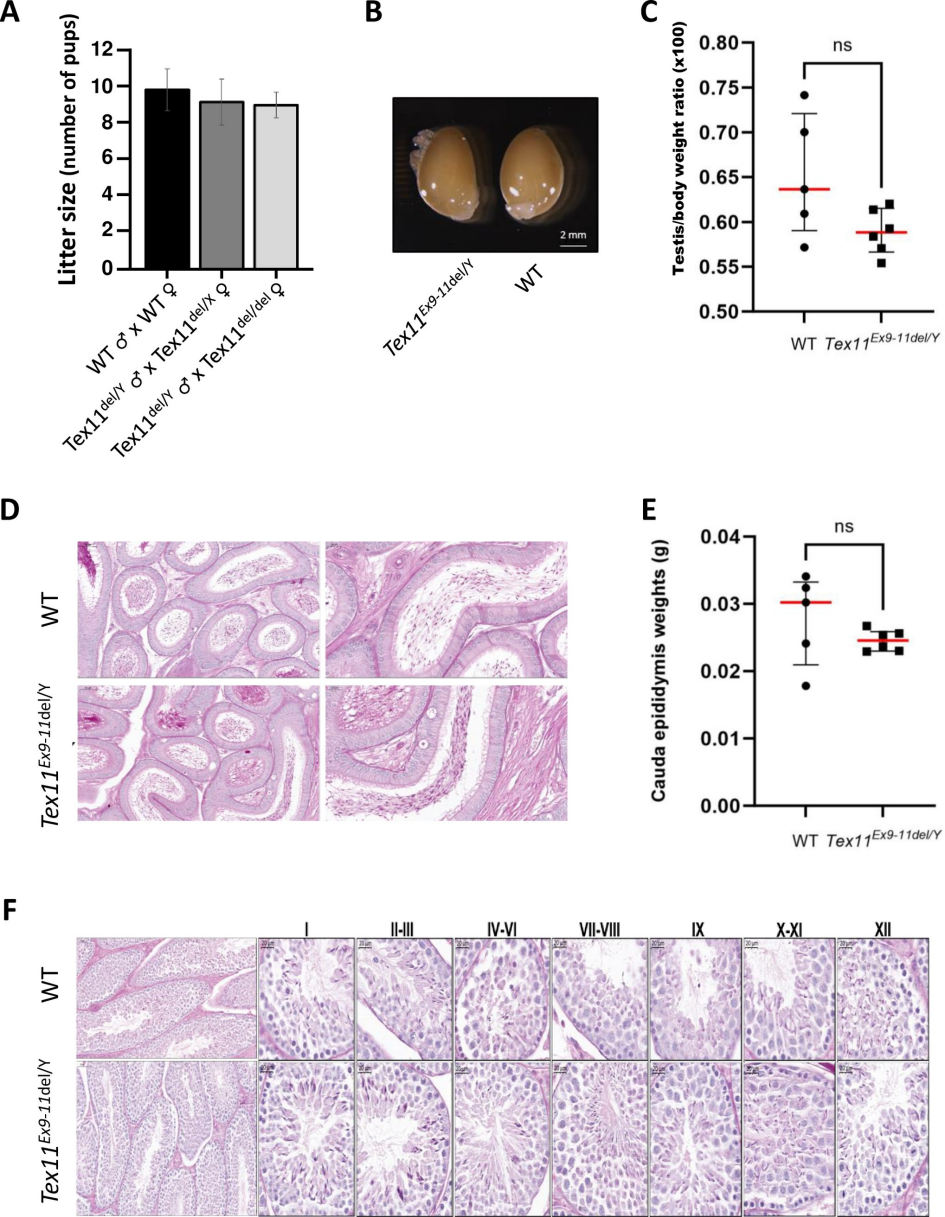
Analysis of spermatogenesis and factors related to male fertility. (A) No significant differences in the average litter size of pups fathered by male WT or *Tex11*^*Ex9-11*del/Y^ mice after mating with female WT, heterozygous, or homozygous mutant mice. (B) Photographs of the testes from two-month-old *Tex11*^*Ex9-11*del/Y^ and WT mice (scale bars: 2 mm). (C) The testicular/body weight ratio of two-month-old mutant vs. WT male mice did not differ significantly, according to a non-parametric Kruskal–Wallis test (p-val = 0.1003). (D) Hematoxylin and eosin stained epididymal sections from 8-week-old WT and *Tex11*^del/Y^ mice. (E) The cauda epididymis weights for WT mice (n = 6) and *Tex11*^*Ex9-11*del/Y^ mice (n = 8) did not differ significantly, according to a non-parametric Kruskal–Wallis test (p-val = 0.2733). The errors bars correspond to the median ± SD. (F) Periodic acid-Schiff-stained sections show the histological structure of the testes from WT and *Tex11*^*Ex9-11*del/Y^ mice. Each image shows a stage in the epithelial seminiferous cycle, which is denoted by Roman numerals at the top of the image. Normal spermatocytes and round and elongated spermatids developed in both control and mutant mice. Scale bars: 50 μm for the two pictures on the left and 20 μm for all the other ones.

Mating tests were performed over a six-month period. The presence of copulatory plugs was used as an indicator of successful mating and reproductive behavior. Surprisingly, *Tex11*^*Ex9-11*del/Y^ males were fertile and therefore produced litters (Fig 2A). Furthermore, we found that the homozygous mutant female *Tex11*^*Ex9-11*del/*Ex9-11del*^ mice were fertile and had litters of much the same size as WT females (Fig 2A). The average litter sizes revealed that all mutant males could sire normal numbers of pups when mated with heterozygous mutant females (*Tex11*^*Ex9-11*del/X^; mean ± standard deviation (SD) litter size: 9.1 ± 2.5) or with homozygous mutant females (*Tex11*^*Ex9-11*del/*Ex9-11del*^; mean ± SD litter size: 9.0 ± 1.4). The equivalent mean ± SD litter size for WT males was 9.8 ± 2.3 (no significant difference, p-val = 0.572) (Fig 2A). Reproduction in mutant mice of both sexes was similar to that in WT animals. Consequently, subsequent crosses were made between females homozygous for the deletion and TEX11 mutant males.

These results suggest that mimicking the deletion found in men with azoospermia did not affect reproduction in mutant mice of both sexes.

### Spermatogenesis and sperm variables in mutant mice

To investigate the impact of *Tex11* deletion on the development of the testicles and spermatozoa, we compared the phenotypes of same-aged WT and mutant male mice. There were no significant differences in testes size, morphology, or weight (Fig 2B and 2C).

To investigate potentially pathologic abnormalities in spermatogenesis in the mutant, we performed histologic analyses. As shown in Fig 2D, the presence of spermatozoa in the lumen of the epididymis duct in *Tex11*^*Ex9-11*del/Y^ mice appears similar to that in WT mice. Furthermore, WT and *Tex11*^*Ex9-11*del/Y^ mice did not differ significantly in terms of the weight and histology of cauda epididymis (Fig 2D and 2E). Lastly, intact spermatogenic tubules and spermatogenic cells at all stages were observed in the testes of *Tex11*^*Ex9-11*del/Y^ mice compared to WT mice (Fig 2F).

**Fig 3:**
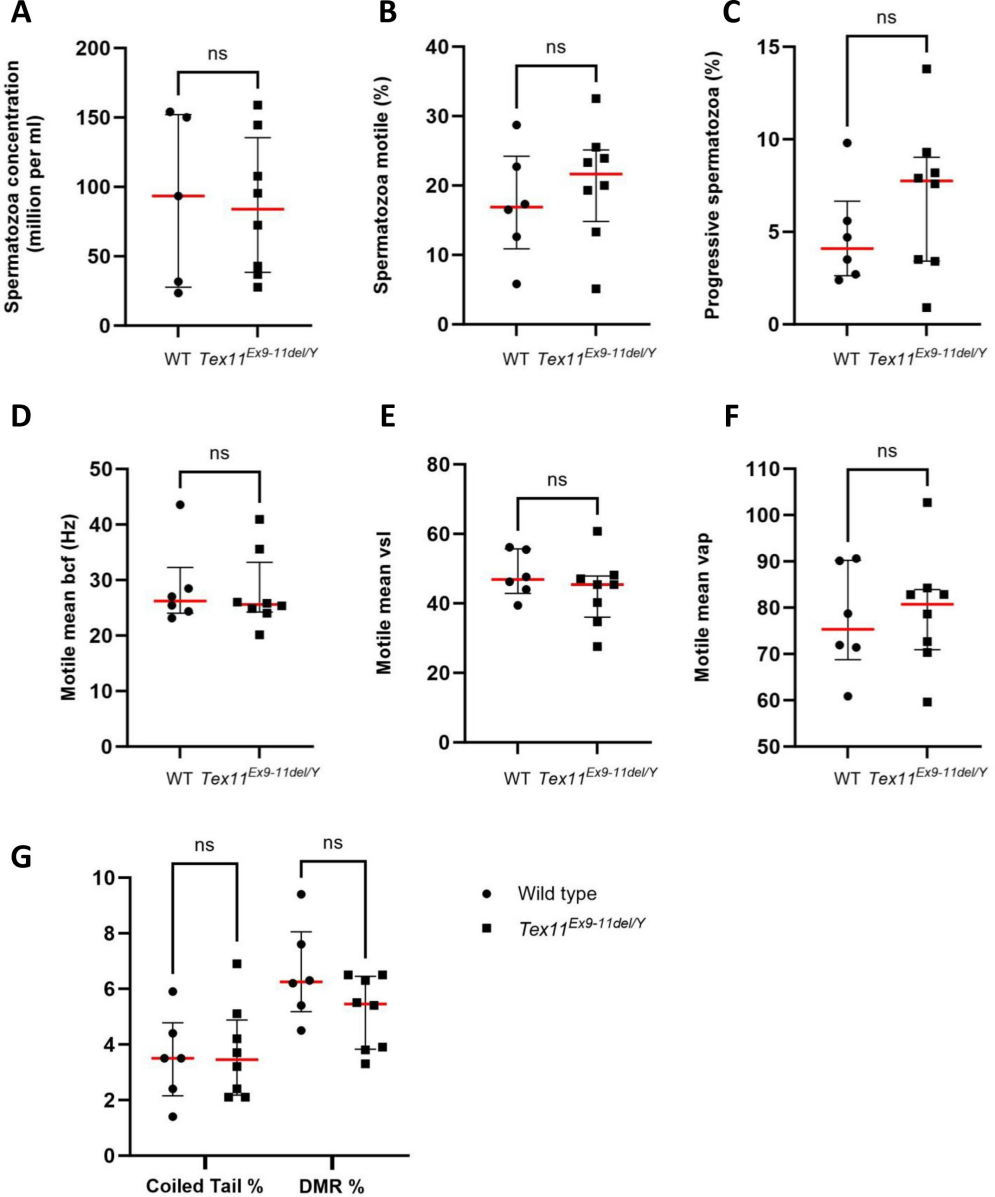
Evaluation of sperm variables. Comparison of sperm-specific variables from WT mice (n = 5 or 6) and *Tex11* mutant (n = 8) mice: (A) Spermatozoa concentration (10^6^/mL), (B) Percentage of motile sperm or (C) progressive spermatozoa (in %) in a computer-assisted sperm analysis. BCF (D), V_SL_ (E), and V_AP_ (F) indicate the beat cross frequency, straight-line velocity, and average path velocity, respectively. (G) Spermatozoa morphological anomalies (% with a coiled tail and % with a distal mid-piece reflex (DMR) abnormality of the sperm tail) in *Tex11*^*Ex9-11*del/Y^ and WT mice. There were no significant differences between WT and mutant mice in any of the variables according to a non-parametric Kruskal–Wallis test ((A) p-val = 0.8836); (B) p-val = 0.3662); (C) p-val = 0.4009); (D) p-val = 0.6985); (E) p-val = 0.4386); (F) p-val = 0.8973); (G) p-val = 0.9484 for coiled trail and p-val = 0.2437 for DMR).

The sperm variables of two-month-old *Tex11* mutant testes were then compared to WT testes of the same age. The mean ± SD sperm concentration in the cauda epididymis was similar for *Tex11*^*Ex9-11*del/Y^ (85.8 ± 49.5 million/mL; n = 8) and control mice (90.6 ± 62.3 million/mL; n = 5); these values (Fig 3A) are in line with the unmodified testis/body weight ratio (Fig 2C). Computer-assisted sperm analysis (CASA) also revealed that the mean ± SD proportion of motile spermatozoa was similar for *Tex11*^*Ex9-11*del/Y^ mice (20.4 ± 8.3; n = 8) and WT mice (17.3 ± 8.00; n = 6) (Fig 3B). Other motility variables (such as the mean count of progressive spermatozoa, the beat cross frequency, and the motile spermatozoa’s mean V_sl_, and V_ap_) were also similar (Fig 3C–3F respectively). Lastly, we confirmed that the *Tex11* deletion did not affect the morphology of mature spermatozoa (Fig 3G).

The CASA results showed that several sperm variables (including the sperm concentration, sperm motility, and sperm morphology) were not impacted by the deletion of exons 9–11 in *Tex11*^*Ex9-11*del/Y^ mutant mice. Overall, mimicking the human deletion of exons 10–12 in the murine *Tex11* gene did not influence the spermatogenesis process in this species.

### A normal transcriptome in Tex11Ex9-11del/Y mouse testes

From the RNA-seq data, we confirmed the absence of transcription of exons 9 to 11 (S3 Fig) in the testis of adult male *Tex11*^Ex9-11*del/Y*^ mice. An analysis of the RNA-seq results revealed that none of the genes were differentially expressed (adjusted p-value (Benjamini–Hochberg) < 0.05 and absolute log2 fold-change > 1) (S3 Table). Taken as a whole, these results demonstrate that the exonic deletion did not affect the expression profile of spermatogenesis genes in the testes.

### Modeling the TEX11 protein

**Fig 4:**
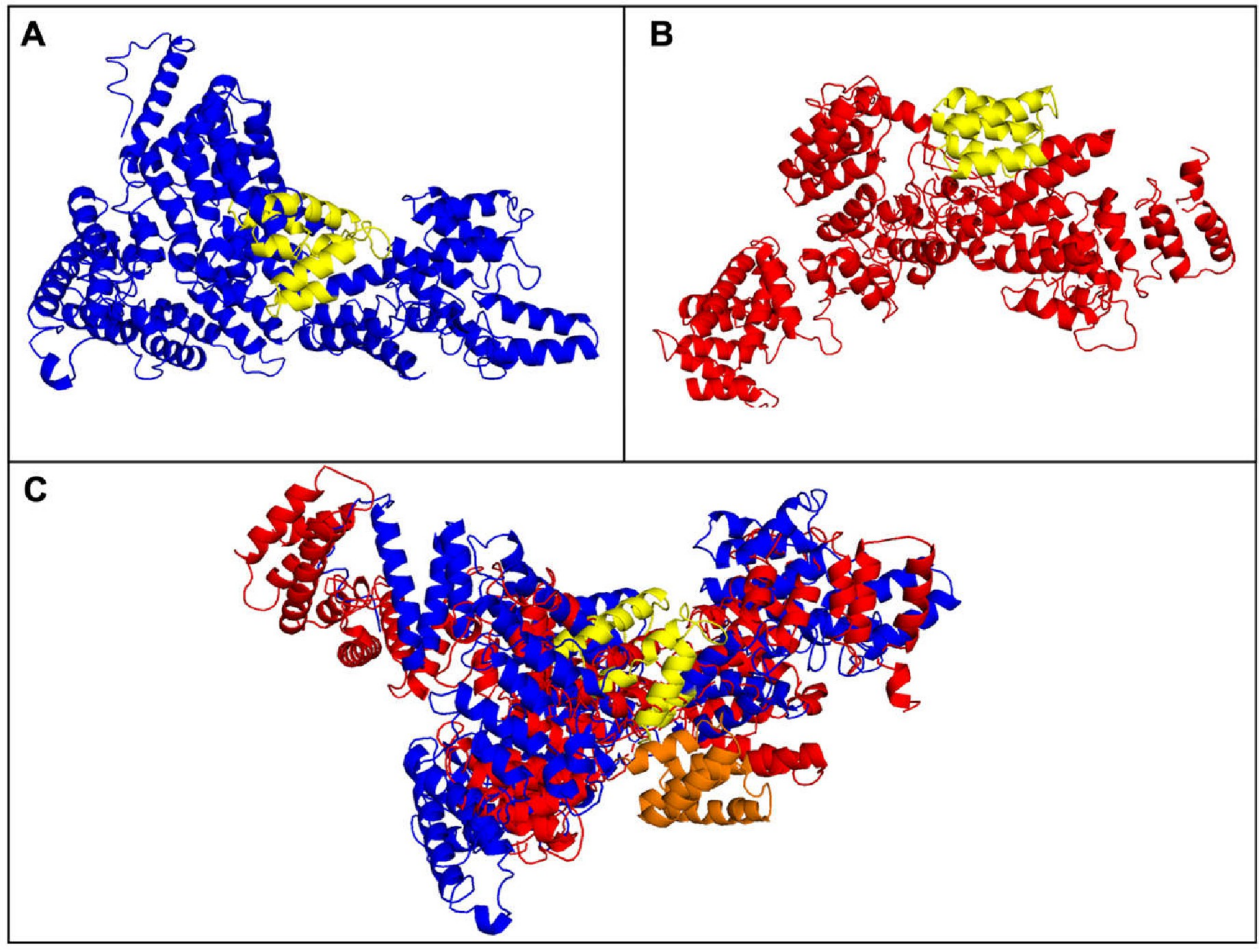
The predicted tertiary structure of TEX11 proteins. Diagrams of tertiary structures of (A) human TEX11 (in blue) (NP_001003811.1) and (B) murine Tex11 (in red) (NP_113561.2). The deleted segments are shown in yellow. (C) Human (in blue) and murine (in red) TEX11 structures are superimposed for easier comparison; the deleted segment of the SPO22 domain is shown in yellow on the human protein and in orange for the murine protein. The tertiary structures were modeled using Phyre2 software and superposed using PyMOL.

We modeled the tertiary structures of human and murine TEX11 (Fig 4) and estimated the potential effects of the exonic deletion on TEX11’s conformation using the Phyre2 and PyMOL algorithms. We found that deletion of exons 10–12 in humans removes approximately four alpha helixes in the resulting human TEX11 protein (Fig 4A), while loss of exons 9–11 in mice removes four and a half alpha helixes in the resulting murine TEX11 protein (Fig 4B). We used PyMOL (https://pymol.org/2/) to superimpose the two 3D structures and compare the conformations (Fig 4C); despite the presence of marked differences overall, the protein’s core (*i*.*e*., the deletion site) was very similar in humans versus mice.

## Discussion

Whole-genome analysis (e.g. chromosome microarray analysis, whole-exome sequencing and whole-genome sequencing) have improved diagnostic and research yields for rare male infertility phenotypes (including NOA) and have identified new candidate genetic factors - especially in patients from consanguineous families (for a review, see [1, 3]). Most candidate genes described to date have been selected based on the phenotype observed in KO mouse models. Spermatogenesis and male infertility are complex, and these overall processes cannot be modeled *in vitro*. As a result, animal models constitute a viable alternative for experimentation.

Given their short reproductive cycle, large litter size, and relatively cheap housing conditions, mice are the most frequently used animal models in biomedical research - including reproductive biology research. Mouse embryos are also relatively easy to manipulate. For these reasons, mouse models (including KO/knock-in, transgenic, and chemically mutagenized mutants) have become popular tools for identifying and then validating disease-associated genes and/or variants.

*Tex11* is a meiosis-specific factor in mice [4]. It encodes a 104-kDa protein with an SPO22 domain and a tetratricopeptide repeat (TPR) pattern that mediates protein-protein interactions and is highly conserved in vertebrates. TEX11 promotes double-strand break (DSB) repair and forms foci on meiotic chromosomes along the synaptonemal complex in spermatocytes and oocytes during the zygotene and early pachytene stages. The protein is expressed abundantly in the cytoplasm of type B spermatogonia and early spermatocytes during the zygotene to pachytene stages. A *Tex11*^-/-^ mouse line (with deletion of exons 3 to 29 out of the 30 in the mouse) showed male infertility due to meiotic arrest, chromosomal asynapsis at the pachytene stage, and reduced crossover formation [4]. In contrast, males and females of another mutant mouse line (with deletion of exon 3, producing a transcript variant in which exon 2 is spliced to exon 4 and that results in a frameshift and a termination codon) are fertile. However, the latter male mice presented a delay in DSB repair in spermatocytes and low levels of crossover formation [5]. At present, *TEX11* is among the most frequently mutated genes in men with NOA [7–10, 12, 16]. Using a 400 K chromosome microarray, a 90-kb hemizygous loss on human chromosome Xq13.2 affecting three exons (10–12) was first identified in two unrelated patients with azoospermia [7]. This loss is predicted to give a 79 amino-acid deletion within the SPO22 domain. Yatsenko *et al*. hypothesized that this human TEX11 deletion is responsible for meiotic arrest and azoospermia. Unlike the human phenotype, we showed that the *Tex11*^*Ex9-11*del^ deletion did not impact gametogenesis, and that *Tex11*^*Ex9-11*del/Y^ male and *Tex11*^*Ex9-11*del/*Ex9-11del*^ female mice were fertile. Moreover, some mutant mouse models with mutations in other testis-specific genes also unexpectedly showed normal fertility (for example [28–30]). In our study, *Tex11*^*Ex9-11*del/Y^ males exhibited normal testis size, sperm concentration, motility, and morphological variables. Furthermore, adult WT and *Tex11*^*Ex9-11*del/Y^ mouse testes had similar transcriptomes.

There are a number of possible explanations for these results:

Firstly, the expressed TEX11 protein lacking 79 amino-acid in the SPO22 domain in *Tex11*-mutant testes might be sufficient for normal function. The truncated TEX11 protein is still expressed in the mutant testes, at levels similar to those seen in control (WT) testes. One can therefore suppose that the exons deleted from the murine *Tex11* gene are not essential for the function of murine TEX11. TEX11 protein expression in patients with these 79 amino acid deletions has not been studied on testicular tissue biopsies [7].

Secondly, the SPO22 domain might not be required for TEX11 function in mice. The SPO22 domain was named by reference to the *Spo22* gene initially described in the yeast *S*. *cerevisiae*. This gene has an ortholog in *Arabidopsis thaliana* (*atZip4*). The yeast Spo22 and atZip4 proteins have similar functions. In-depth studies of Spo22 and atZip4 mutants has shown that Zip4/Spo22 proteins help to stabilize chromosome pairing beyond the pachytene stage and promote stable connections and thus adequate chromosome disjunction. The Spo22 protein is also part of the ZMM group involved in the formation of the crossing-over and synaptonemal complex [31]. The Zip4/Spo22 mutant in *S*. *cerevisiae* exhibits delayed meiotic progression and reduced spore viability and crossover formation, due to a lack of synaptonemal complex formation [32]. In contrast to the phenotype observed in *S*. *cerevisiae*, the atZip4 mutants in *A*. *thaliana* show regular pairing of homologous chromosomes before metaphase I but a low frequency of crossing over [33]. Hence, this group of meiosis-specific proteins (including yeast Spo22, plant ZIP4 and, through its SPO22 domain, mammalian TEX11) appears to have a crucial role in crossover formation and meiotic chromosome segregation. However, there are no data on the specific role of the TEX11 SPO22 domain in mice. To the best of our knowledge, our study is the first to have analyzed TEX11’s role in mouse reproduction through the deletion of a large proportion of the SPO22 domain. We therefore assume that this domain is not necessary for meiosis and thus fertility in mice. Even without 79 amino acids of the SPO22 domain, the TEX11 protein is sufficient for normal meiotic function.

Thirdly, the exonic deletion might not be responsible for the azoospermia phenotype. The deletion of *TEX11* exons 10 to 12 was first identified in two azoospermic patients: one with mixed testicular atrophy, and the other with meiotic arrest [7]. Yatsenko *et al*. suggested that the partial deletion of *TEX11* was responsible for the men’s azoospermia. However, neither the presence of a mutant TEX11 protein nor its location were analyzed. Furthermore, the two patients had different testicular phenotypes: one showed a mixed phenotype, with partial spermatogenesis in some seminiferous tubules. The presence of mature germ cells in one of the deletion carriers indicates that the *TEX11* deletion may not lead to complete meiotic arrest, and that the SPO22 domain is not essential for gametogenesis in humans and mice. In such a case, the deletion should be reclassified as a variant of unknown significance.

Fourthly, the infertility phenotype might depend on the effect of the human variant on TEX11 protein expression. With the exception of the deletion of exons 10–12 [7], few of the *TEX11* gene variants described in the literature (S4 Table: c.358C>T [9]; c.1051G>T [12], c.1254dupA [12], c.857delA [12]; c.1291C>T [11]; c.84-651del [15]; c.792+1G>A [7]; c.776C>T [8]; and c.1303Ins(TT) [8]) affect the SPO22 protein domain. Some of these mutations affected the expression of TEX11 protein, while others had no effect or were not analyzed (S4 Table). For instance, mice carrying the frameshift insertion (c.1303Ins(TT)) displayed meiotic arrest and a low amount of TEX11 protein [8]. c.1303Ins(TT)) resulted in a stop codon at amino acid 446, which in turn led to deletion of the last 948 amino acids (including the 14 last amino acids of the SPO22 domain and the rest of the protein). Consequently, the mice were sterile. Considering the large number of functional spermatogonial stem cells in the mouse testis, these cells were rectified using the CRISPR/Cas9-mediated homology-directed repair. This experiment successfully restored fertility in the recipient mice [18]. Furthermore, transfected cells with mutations showed either low TEX11 protein expression (c.1051G>T, c.1254dupA, and c.857delA) or the complete absence of TEX11 protein (c.298delG, which does not affect the SPO22 domain) [12]. Lastly, the c.1291C>T mutation in the SPO22 domain induces a premature stop codon, which might explain the patient’s infertility [11]. In view of our results and the literature data on *TEX11* mutations, one can hypothesize that genetic alterations in the SPO22 domain lead to infertility only if the variant lead to the total absence of protein expression. Further functional analyses are needed to confirm whether mutations like c.84-651del, c.792+1G>A, and c.776C>T affect TEX11 expression.

Fifthly, the murine and human proteins might have different 3D conformations. Yatsenko *et al*. modeled the human TEX11 protein and showed that alpha helixes accounted for more than 70% of the secondary structure [7]. The researchers also evaluated the possible effect of the exonic deletion on the human TEX11 tertiary structure; they estimated that the deletion of exons 10 to 12 removes nearly three alpha helixes from the SPO22 domain. In our study, modeling revealed likely differences in the tertiary structure of the murine and human TEX11 proteins. This is further supported by the dot matrix analysis, which showed that only 56% of regions were similar when comparing the human and the murine TEX11 proteins (S1C Fig). Accordingly, we speculate that spermatogenesis in the two species involves different TEX11 protein interactions and mechanisms. Apart from the SPO22 domain, the TEX11 protein contains TPRs that mediate protein-protein interactions [34]. According to the TPR prediction with TPRpred (https://toolkit.tuebingen.mpg.de/tools/tprpred), the murine *Tex11* transcript isoform 1 has eight TPRs motifs and the human TEX11 transcript isoform 1 has five. The exonic loss eliminates two of these TPR domains in mice but only one in humans. We can assume that while six TPR motifs in mice may be sufficient for the protein activity, four TPRs in humans might not be. The loss of TPRs motifs might be why the phenotype is less damaging in mice than in humans. TPR-domain-containing proteins are known to have their role in fertility and the assembly of cilia and flagella [35–37].

Lastly, the truncated protein’s effects might be influenced by the genetic background. A more severe phenotype might emerge in backgrounds other than the one studied here. The background hypothesis was mentioned by Tran and Schimenti [38], who had had similar results with a mouse model of a putative infertility allele DMC1^M200V^. DMC1^M200V/M200V^ male and female mice were fully fertile and had no reproductive or meiotic abnormalities. Some *Tex11* mouse mutant models showed poor reproductive phenotypes [4, 8]; others showed no reproductive abnormalities, and both male and female mutant mice were fertile [5]. The various phenotypes in *Tex11* mutant mouse models might be due to the genetic background. Many studies have shown that mutant infertility phenotypes can depend on the genetic background [39–41]. For example, mutations in the *Usp26* gene caused sterility or subfertility in mutant males backcrossed on a DBA/2 background but not on a C57BL/6 background [39]. An additional example is the Ter point mutation, which was mapped on the RNA-binding protein DND. This mutation induces a premature stop codon, which ultimately leads to severe germ cell loss in both sexes. However, in males, germ cells fail to undergo mitotic arrest, resulting in a markedly elevated incidence of testicular teratomas, which is contingent upon the genetic background [42, 43]. The genetic background also influences female fertility as recently highlighted [44]. The abnormalities resulting from leptin deficiency give rise to a complex phenotype, which is dependent on the genetic background [45]. Additionally, the reproductive performance of mutants is contingent upon the specific strain under consideration. A mutation in the βB2-crystallin gene, which is expressed in the gonads, has been observed to result in subfertility in mice. This subfertility is attributed to defects in sperm and egg production. However, the C57Bl/6 genetic background did not exhibit any defects in reproductive function [46]. Thus we cannot formerly exclude that the analyzed TEX11 truncation would result in a male fertility phenotype if a different genetic background were used.

In conclusion, the *TEX11* gene is highly conserved from yeast to mammals and is thought to be essential for murine and human spermatogenesis. Here, we generated a line of mice carrying a *Tex11* mutation that mimicked the *TEX11* mutation found in two azoospermic men. The mutant male and female mice were fully fertile. The structural defect in the murine TEX11 protein was not sufficient to cause the defect of azoospermia. Our study highlights the complexity of genetic interactions and biological mechanisms governing fertility, and shed light on significant differences between animal models and humans. Further research is needed to elucidate the underlying mechanisms responsible for this discrepancy, which could eventually lead to new insights for the treatment of human infertility.

## Supporting information

S1 Fig Comparison of *TEX11* gene in the human vs. the mouse. (A) Diagram showing the structures of the first five exons and last three exons of the human *TEX11* gene and the first four exons and last three exons of the murine *Tex11* gene. (B-C) A dot matrix view showing regions of similarity (based on the BLAST results) for (B) human *TEX11* mRNA (NM_001003811) (on the x axis) and mouse *Tex11* mRNA (NM_031384) (on the y axis) and (C) the human TEX11 protein (NP_001003811) (on the x axis) and the mouse TEX11 protein (NP_113561) (on the y axis). The numbers correspond to the nucleotide bases (B) or amino acids (C) in the sequences. Alignments are shown as lines. The number of lines shown in the plot is the same as the number of alignments found by BLAST (BLASTn in (B) and BLASTp in (C)).
(PDF)

S2 Fig MultAlin sequence alignment of human and murine deleted exons. (A) Alignment of human exon 10 (NM_001003811) and mouse exon 9 (NM_031384). Percentage identity: 75.6%. (B) Alignment of human exon 11 (NM_001003811.2) and mouse exon 10 (NM_031384). Percentage identity: 83.6%. (C) Alignment of human exon 12 (NM_001003811.2) and mouse exon 11 (NM_031384). Percentage identity: 70.8%. The sequences were aligned using the MultAlin website (http://multalin.toulouse.inra.fr/multalin/). Percentage identity values were calculated with the EMBOSS Matcher tool from the EMBL-EBI (https://www.ebi.ac.uk/Tools/psa/).
(PDF)

S3 Fig Deletion of exons 9, 10, and 11 of the murine Tex11 gene. Transcription of the reverse strand of chromosome X between exons 8 to 13 in the Tex11 gene, with the RNA-seq coverage (Integrative Genomics Viewer (IGV) representation from BigWig files of strand-specific RNA-seq data) in WT (top blue tracks, n = 3) and Tex11Ex9-11del/Y (bottom tracks, n = 9) adult testes.
(PDF)

S1 Table A list of the primers used in this study (genotyping and sgRNAs). (PDF)

S2 Table A comparison of murine *Tex11* mRNA (NM_031384) and human TEX11 mRNA (NM_001003811.1). Regions of non-similarity (based on the BLAST results) are shaded. Human exons 10–12 are homologous to murine exons 9–11.
(PDF)

S3 Table Result table from a DESeq2 analysis from (Tex11^Ex9-11del/Y^ gonads versus control testes). (XLSX)

S4 Table List of human TEX11 variants identified in the literature on NOA. Intronic mutations are not listed. Mutations are mapped to isoform 1 (NM_001003811).
(XLSX)
