# Supplementary figures and images for: A partial deletion within the meiosis-specific sporulation domain SPO22 of Tex11 is not associated with infertility in mice
Source: PLoS One. 2024 Sep 4;19(9):e0309974. doi: 10.1371/journal.pone.0309974 (PMC11373865; doi:10.1371/journal.pone.0309974)

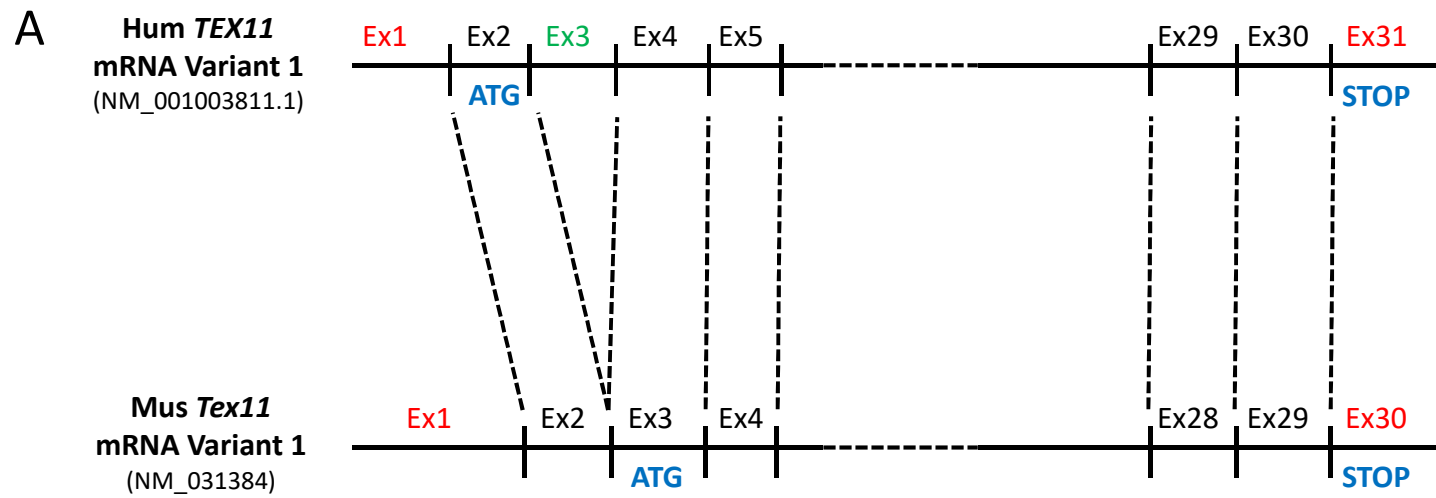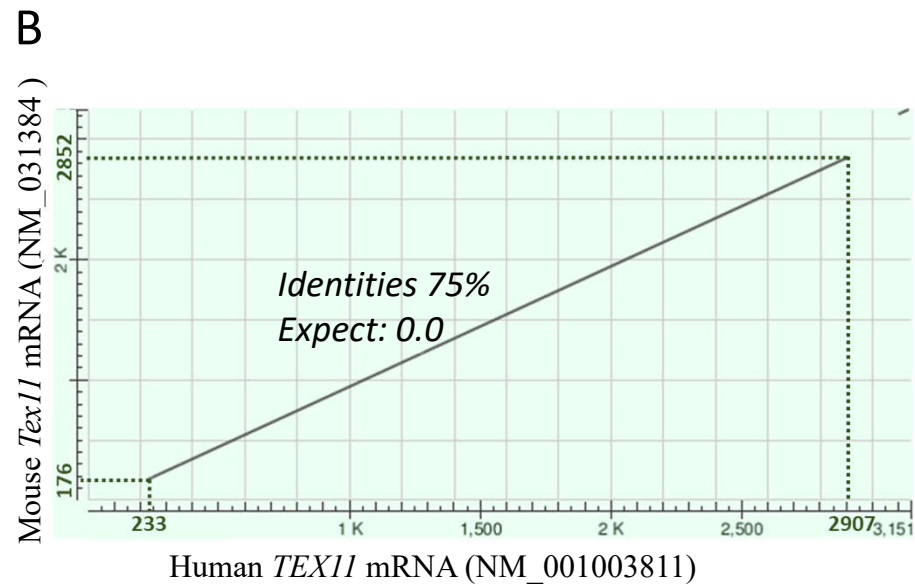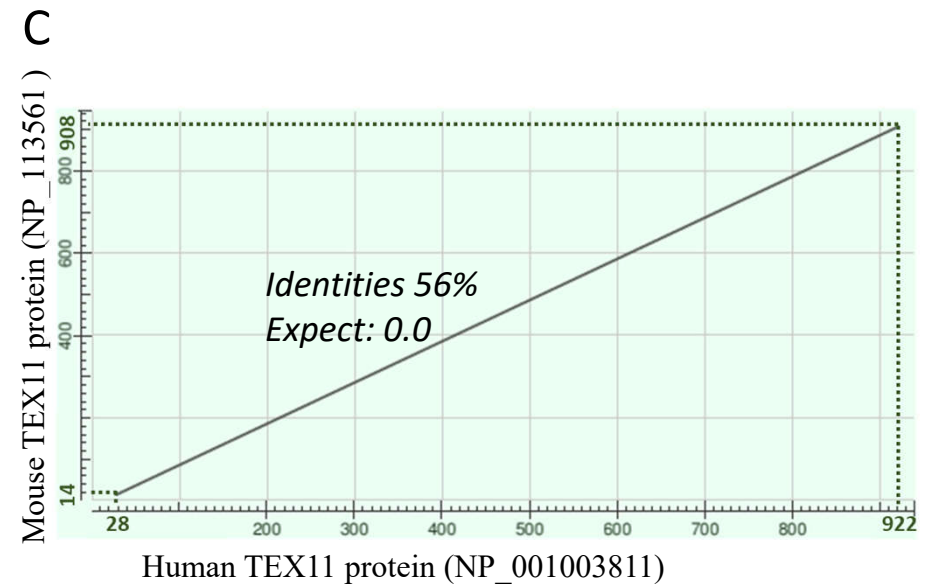

Supplement: S1 Fig — (A) Diagram showing the structures of the first five exons and last three exons of the human TEX11 gene and the first four exons and last three exons of the murine Tex11 gene. (B-C) A dot matrix view showing regions of similarity (based on the BLAST results) for (B) human TEX11 mRNA (NM_001003811) (on the x axis) and mouse Tex11 mRNA (NM_031384) (on the y axis) and (C) the human TEX11 protein (NP_001003811) (on the x axis) and the mouse TEX11 protein (NP_113561) (on the y axis). The numbers correspond to the nucleotide bases (B) or amino acids (C) in the sequences. Alignments are shown as lines. The number of lines shown in the plot is the same as the number of alignments found by BLAST (BLASTn in (B) and BLASTp in (C)). (PDF) [file pone.0309974.s001.pdf]

Chromosome X

Tex 11 ←

Mouse Refseq  
Genes (mm10)

Exons 13 12 11 10 9 8

100 980 kb

100 990 kb

101 000 kb

101 010 kb

101 020 kb

WT1

WT2

WT3

KO1

KO2

KO3

KO4

KO5

KO6

KO7

KO8

KO9

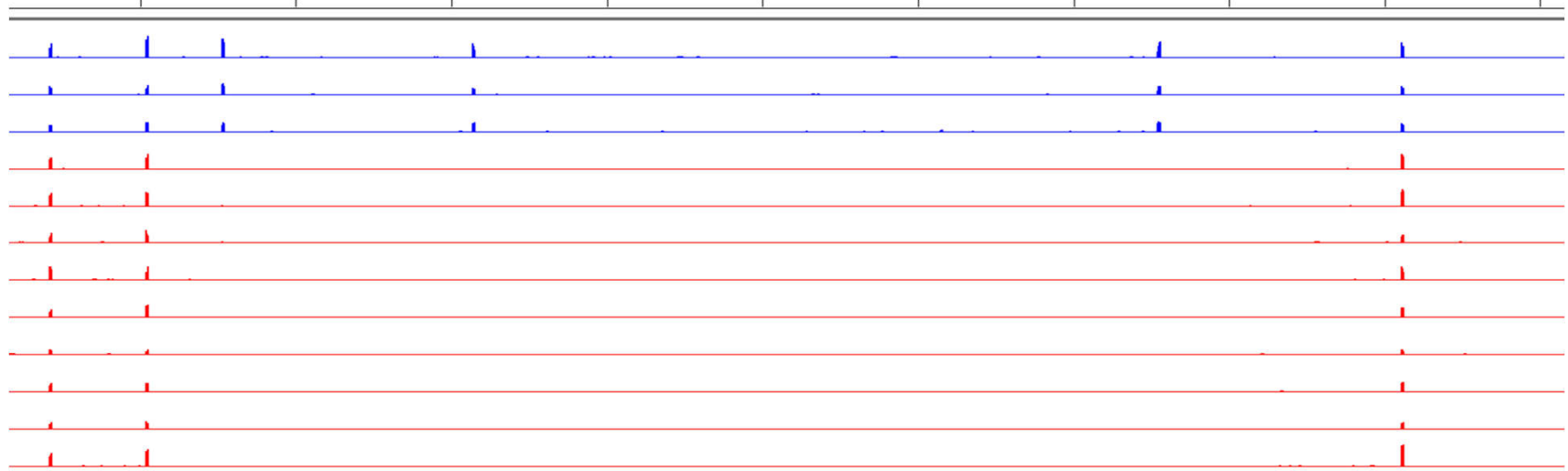

Supplement: S3 Fig — Transcription of the reverse strand of chromosome X between exons 8 to 13 in the Tex11 gene, with the RNA-seq coverage (Integrative Genomics Viewer (IGV) representation from BigWig files of strand-specific RNA-seq data) in WT (top blue tracks, n = 3) and Tex11Ex9-11del/Y (bottom tracks, n = 9) adult testes. (PDF) [file pone.0309974.s003.pdf]
