# Supplementary material for: A partial deletion within the meiosis-specific sporulation domain SPO22 of Tex11 is not associated with infertility in mice
Source: PLoS One. 2024 Sep 4;19(9):e0309974. doi: 10.1371/journal.pone.0309974 (PMC11373865; doi:10.1371/journal.pone.0309974)
Supplement: S2 Fig — (A) Alignment of human exon 10 (NM_001003811) and mouse exon 9 (NM_031384). Percentage identity: 75.6%. (B) Alignment of human exon 11 (NM_001003811.2) and mouse exon 10 (NM_031384). Percentage identity: 83.6%. (C) Alignment of human exon 12 (NM_001003811.2) and mouse exon 11 (NM_031384). Percentage identity: 70.8%. The sequences were aligned using the MultAlin website (http://multalin.toulouse.inra.fr/multalin/). Percentage identity values were calculated with the EMBOSS Matcher tool from the EMBL-EBI (https://www.ebi.ac.uk/Tools/psa/). (PDF) [file pone.0309974.s002.pdf]

A

```
             1        10        20        30        40        50        60        70        80    86
             |--------+---------+---------+---------+---------+---------+---------+---------+-----|
Hum_exon10   ACTTCAAGTCTTCATCATCTCTGTTACAACTTTGGAGTAGAAACCCAGAAGAATAATAAATATGAAGAAAGTTCTTTCTGGCTTAG
Mus_exon9    ACAAAATATCTTCATGTACTCTGTTACAACCTTGGCATAGAAGCAAGCAAGCGGAATAAATACAAAGAGAGTTCATTCTGGCTTGG
Consensus    ACaaaAaaTCTTCATcaaCTCTGTTACAACcTTGGaaTAGAAaCaaacAAGaagAATAAATAcaAAGAaAGTTCaTTCTGGCTTaG
```

B

```
             1        10        20        30        40        50   55
             |--------+---------+---------+---------+---------+----|
Hum_exon11   CCAAAGCTATGATATTGGGAAGATGGATAAGAAATCTACTGGGCCAGAAATGCTG
Mus_exon10   CCAAAGCTATGAAATTGGGAAGATGGATAGGCGTTCTGTTGAGCCACAAATGCTG
Consensus    CCAAAGCTATGAaATTGGGAAGATGGATAaGaaaTCTacTGaGCCAcAAATGCTG
```

C

```
             1        10        20        30        40        50        60        70        80        90    96
             |--------+---------+---------+---------+---------+---------+---------+---------+---------+-----|
Hum_exon12   GCTAAAGTTCTACGGCTATTAGCCACGAATTATTTGGATTGGGATGACACCAAATATTATGATAAGGCTCTCAATGCTGTAAACCTAGCAAACAAG
Mus_exon11   GCTAAAACGCTGCGGTTACTAGCCACTATTTATTTGAATTGTGGTGGCGAAGCATATTATACCAAGGCCTTCATTGCTATACTCATTGCAAACAAG
Consensus    GCTAAAacgCTaCGGcTAcTAGCCACgAaTTATTTGaATTGgGaTGaCaaaaaATATTATaacAAGGCccTCAaTGCTaTAaaCaTaGCAAACAAG
```
