# Supplementary material for: A partial deletion within the meiosis-specific sporulation domain SPO22 of Tex11 is not associated with infertility in mice
Source: PLoS One. 2024 Sep 4;19(9):e0309974. doi: 10.1371/journal.pone.0309974 (PMC11373865; doi:10.1371/journal.pone.0309974)
Supplement: S1 Table — (PDF) [file pone.0309974.s004.pdf]

Supplementary Table S1: List of primers used in this study (genotyping and gRNAs).

Genotyping PCR primers				Size
**Primer name**	**5'-Forward-3'**	**Primer name**	**5'-Reverse-3'**	
Tex11_64241F	GTGCACCTTTCTGGGTGTTT	Tex11_64843R	GCACTTTTCAGGCAGGACTC	622 bp for WT
Tex11_50129F	ACCCTGGTTGATTGTTTCCA	Tex11_83418R	GAACACCGATGTGTGTGTGAT	600 bp for Tg

CRISPR/Cas9 guide RNA	
gRNAs_5'_ex9-1 (fwd)	3'-CTCACTAGTTCATAGGTATC *(AGG)*-5'
gRNAs_5'_ex9-2 (rev)	3'-TCAACAGGACCTAATAGTAC *(AGG)*-5'
gRNAs_3'_ex11-1 (rev)	3'-AGATAACGCACATGCGGCTG *(AGG)*-5'
gRNAs_3'_ex11-2 (fwd)	5'-CCTAGTAACCTAATAGCACC *(TGG)*-3'

*PAM sequences are given in brackets
