# Supplementary material for: A partial deletion within the meiosis-specific sporulation domain SPO22 of Tex11 is not associated with infertility in mice
Source: PLoS One. 2024 Sep 4;19(9):e0309974. doi: 10.1371/journal.pone.0309974 (PMC11373865; doi:10.1371/journal.pone.0309974)
Supplement: S2 Table — Regions of non-similarity (based on the BLAST results) are shaded. Human exons 10–12 are homologous to murine exons 9–11. (PDF) [file pone.0309974.s005.pdf]

Supplementary Table S2 Comparison of murine (NM_031384) and human (NM_001003811.1) TEX11 mRNAs.
Regions of non-similarity based on BLAST results are shaded. Human exons 10-12 are homologous to murine exons 9-11.

	Mouse *Tex11* mRNA variant 1 (NM_031384)		Human *TEX11* mRNA variant 1 (NM_001003811.1)	
EXONS	1	1-127	1	1-64
	2	128-189	2	65-122
			3	supernumerary
	3	190-311	4	245-366
	4	312-396	5	367-451
	5	397-476	6	452-531
	6	477-557	7	532-612
	7	558-677	8	613-732
	8	678-758	9	733-813
	9	759-844	10	814-899
	10	845-899	11	900-954
	11	900-995	12	955-1050
	12	996-1077	13	1051-1132
	13	1078-1156	14	1133-1211
	14	1157-1308	15	1212-1363
	15	1309-1394	16	1364-1449
	16	1395-1532	17	1450-1587
	17	1533-1635	18	1588-1690
	18	1636-1760	19	1691-1815
	19	1761-1846	20	1816-1901
	20	1847-1903	21	1902-1958
	21	1904-1944	22	1959-1999
	22	1945-2031	23	2000-2086
	23	2032-2102	24	2087-2157
	24	2103-2219	25	2158-2274
	25	2220-2292	26	2275-2347
	26	2293-2442	27	2348-2497
	27	2443-2551	28	2498-2606
	28	2552-2672	29	2607-2727
	29	2673-2837	30	2728-2892
	30	2838-3250	31	2893-3151
